# Exploration of the Association between Obesity and Semen Quality in a 7630 Male Population

**DOI:** 10.1371/journal.pone.0119458

**Published:** 2015-03-30

**Authors:** Chih-Wei Tsao, Chin-Yu Liu, Yu-Ching Chou, Tai-Lung Cha, Shih-Chang Chen, Chien-Yeh Hsu

**Affiliations:** 1 Division of Urology, Department of Surgery, Tri-Service General Hospital, National Defense Medical Center, Taipei, Taiwan; 2 Department of Nutritional Science, Fu Jen Catholic University, New Taipei City, Taiwan; 3 School of Public Health, National Defense Medical Center, Taipei, Taiwan; 4 Graduate Institute of Patent, National Taiwan University of Science and Technology, Taipei, Taiwan; 5 Department of Information Management, National Taipei University of Nursing and Health Sciences, Taipei, Taiwan; 6 Master Program in Global Health and Development, Taipei Medical University, Taipei, Taiwan; McMaster University, CANADA

## Abstract

This study aimed to explore the association between body mass index (BMI), other anthropometric indexes and semen quality in a general male population in Taiwan. In this cross-sectional cohort study, the study cohort consisted of 7941 healthy male individuals aged 18 years or older who participated in a standard medical screening program run by a private firm from January 2008 to May 2013. Semen parameters including sperm concentration (SC), total sperm motility (TSM), progressive motility (PRM), and normal sperm morphology (NSM) were recorded. Anthropometric indexes including BMI, waist circumference (WC), hip circumference (HC), waist-to-hip ratio (WHR), waist-to-height ratio (WHtR) and body fat percentage were measured. A total of 7630 men were enrolled for the final analysis, of whom 68.5% had a normal weight distribution and 31.4% were overweight or obese. Total sperm motility, progressive motility, normal sperm morphology and sperm concentration showed a statistically linear decline with increasing age (*p* < 0.001, *p* < 0.001, *p* < 0.001 and *p* = 0.004). Sperm concentration showed a significantly negatively linear association with BMI (*p* = 0.005), and normal sperm morphology showed an inverse association with BMI and waist-to-height ratio (*p* < 0.001 and *p* = 0.004). The prevalence of abnormal total sperm motility, progressive motility, normal sperm morphology and sperm concentration increased with increasing age (*p* = 0.011, *p* < 0.001, *p* < 0.001 and *p* = 0.002). Lower normal sperm morphology and sperm concentration were associated with increasing body adiposity (*p*<0.05). No relationship between obesity and sperm motility was identified.

## Introduction

It is well-known that overweight and obese women are more likely to experience ovulatory and menstrual disorders, which consequently lead to delayed fertility [1]. A cross-sectional study of 47835 Danish couples reported that overweight and obese women are at an increased risk of subfertility, with a statistically significant trend of a prolonged time to pregnancy of more than 12 months [2]. However, an association between high adiposity and subfertility has not been clearly demonstrated in men. Several studies have reported inverse correlations of sperm concentration and total sperm count with obesity when assessed by body mass index (BMI) [3], central adiposity according to waist circumference (WC) [4] and hip circumference (HC) [5]. Meanwhile, a systemic review by meta-analysis found no association between a high BMI and male semen parameters [6]. In addition, another study explored the relative risks for semen parameters being below World Health Organization reference limits in overweight and obese men, and found no significant associations between BMI and semen parameters with the exception of normal sperm morphology [7]. Furthermore, a recent meta-analysis of 21 studies with 13,077 subjects reported a J-shaped association between BMI and abnormal total sperm count, and also that overweight and obesity were associated with an increased prevalence of azoospermia or oligospermia [8]. Palmer et al. ever concluded from a literature review that the relationship between obesity and semen quality was conflicting [9]. Colaci group [10] reported that fertilization rate was higher among obese men than among normal weight in conventional IVF cycle. No statistically significant associations were identified between male BMI and proportion of poor-quality embryos. Moreover the largest subjects of single institute including 10,665 males declared that increasing BMI being associated with decreased semen quality, affecting volume, concentration and motility recently [11].

In order to identify factors affecting semen quality, this study aimed to assess the relationship between obesity and sperm parameters in a large study cohort in Taiwan, and to investigate the separate anthropometric indexes and age effects on male fertility.

## Materials and Methods

This cross-sectional study consisted of 7941 healthy Taiwanese male individuals aged 18 years or older who participated in a standard medical screening program run by a private firm (MJ Health Management Institution, Taipei, Taiwan) between January 2008 and May 2013. The firm attracted paying participants from all over Taiwan because of its known quality services, operational efficiency, and key facilities that were easily accessible. Membership to the program was required, with discounts in examination fees offered for people with a large-size family or related individuals and for regular members who came back for repeated examinations in subsequent years, and these incentives succeeded in attracting and sustaining a large number of customers. Each participant signed a consent form authorizing MJ Health Management Institution to process the data generated from medical screening. Ethical reviews and assessments were processed and approved by MJ Institutional Review Boards in Taiwan. Data related to individual identification were removed, ensuring the anonymity of each individual during the entire study process.

After excluding those with incomplete records, a total of 7630 subjects were enrolled into the study. Semen sample was collected via masturbation following 3 days of abstinence using home collection kits, and the sample was sent to the laboratory for analysis within one hour. Four dependent semen parameters including sperm concentration (SC), total sperm motility (TSM), progressive motility (PRM) and normal sperm morphology (NSM) were recorded. Sperm concentration was evaluated by hemocytometer (Improved Neubauer; Hauser Scientific, Inc., Horsham, PA, USA). Samples were diluted in a solution of 0.6 M NaHCO3 and 0.4% (v/v) formaldehyde in distilled water. Sperm motility was classified as progressive motility (WHO class A+B) and total sperm motility (WHO class A+B+C) [12]. Briefly, a 10 ml of well-mixed semen was placed on a clean glass slide that had been kept at 37°C and covered with a 22 × 22 mm coverslip. The preparation was placed on the heating stage of a microscope at 37°C and immediately examined at ×400 magnification. Morphology was assessed using strict criteria [13].

In addition, we recorded anthropometric indexes [BMI, waist circumference, hip circumference, waist-to-hip ratio (WHR), waist-to-height ratio (WHtR) and body fat percentage] and biochemistry data to analyze the relationships between obesity or derivative markers and semen quality. BMI was calculated as weight in kilograms divided by height in meters squared. Waist circumference was obtained from the mid-point between the iliac crest and the costal margin, and hip circumference was measured at the widest point around the greater trochanter. Waist-to-hip ratio was calculated as waist circumference divided by hip circumference, and waist-to-height ratio was calculated as waist circumference divided by height. Measurements of percentage body fat were performed by the bioelectrical impedance analysis (BIA) technique by using a body-composition analyzer. A venous blood sample was taken after at least an 8-h fast for the measurement of plasma triglycerides, total cholesterol, low-density lipoprotein (LDL) cholesterol and high-density lipoprotein (HDL) cholesterol. These assays were performed on a HITACHI 7150.

As Asian populations have different criteria of obesity as compared with Western populations [14–16], we used quartiles to evaluate the relationships between the anthropometric indexes and semen quality. Differences in the sperm parameters by varying degrees of obesity as assessed by the different anthropometric indexes were compared by one-way analysis of variance (ANOVA). The *p* values of statistics after adjusting confounding factors were calculated by the linear regression models.

Abnormal sperm parameters were defined as sperm concentration < 15 M/ml, total sperm motility < 40%, progressive motility < 32% and normal sperm morphology < 30%. The frequencies of abnormal sperm parameters in each quartile were compared by the chi-square test. We assessed the relationships between adiposity and age with semen parameters by first conducting logistic trend analysis, and then estimating the odds ratios (ORs). Moreover, the topics of smoking have been hot and popular in the public health, thus we applied a questionnaire to assess the relationship associated with male infertility. According to the questionnaire, the smoking duration was assessed as “0”: no smoking, “1”: smoking < 1 year, “2”: 1 year to < 3 years, “3”: 3 years to < 5 years, “4”: 5 years to < 10 years, “5”: 10 years to < 20 years, “6”: 20 years or more. We then explored the association between smoking and semen quality by the quartile method (dividing [“0”], [“1”+”2”], [“3”+”4”], [“5”+”6”]). To avoid the risk of false positive result from multiple testing between potential correlated factors and 4 groups of age, BMI, anthropometric measurements (waist and hip circumference, waist-to-hip and waist-to-height ratio, and body fat) and smoking duration by quartiles (in Table 1 and 2). The one-way ANOVA with Bonferroni post hoc procedures was considered. First, we identified the total number of different statistical tests were equal to 6. Subsequently, we calculated the Bonferroni corrected p-value threshold in this present study from 0.05 to 0.008 (P corrected = 0.05/6 = 0.008) and values of p<0.008 were considered statistically significant. All analyses were conducted using SPSS statistical software (version 13.0, SPSS Inc, Chicago, IL).

**Table 1:** Characteristics of the general male population (*n* = 7630).

Characteristics	BMI < 21.60 (*n* = 1907) Mean (95% CI for mean)	BMI: 21.60–23.48 (*n* = 1908) Mean (95% CI for mean)	BMI: 23.49–25.61 (*n* = 1908) Mean (95% CI for mean)	BMI ≥ 25.62 (*n* = 1907) Mean (95% CI for mean)	Total (*n* = 7630) Mean (95% CI for mean)	*p* ^a^ ^,^ ^c^
**Age (years)**	30.83 (30.64, 31.02)	31.78 (31.58, 31.98)	32.16 (31.95, 32.38)	32.21 (31.99, 32.43)	31.75 (31.64, 31.85)	< 0.001
**Triglyceride**	87.55 (85.27, 89.83)	106.81 (103.98, 109.64)	127.90 (124.23, 131.56)	153.93 (148.83, 159.04)	119.06 (117.16, 120.95)	< 0.001
**Cholesterol**	179.18 (177.73, 180.62)	188.62 (187.17, 190.07)	192.64 (191.13, 194.16)	198.11 (196.55, 199.68)	189.64 (188.87, 190.40)	< 0.001
**HDL**	55.22 (54.63, 55.81)	51.13 (50.61, 51.65)	48.04 (47.54, 48.53)	46.17 (45.71, 46.64)	50.18 (49.91, 50.46)	<0.001
**LDL**	106.65 (105.33, 107.97)	116.63 (115.26, 117.99)	119.97 (118.57, 121.37)	122.93 (121.51, 124.35)	116.47 (115.77, 117.17)	< 0.001
**Cholesterol ratio**	3.38 (3.34, 3.42)	3.84 (3.80, 3.89)	4.16 (4.11, 4.21)	4.44 (4.38, 4.49)	3.95 (3.92, 3.97)	< 0.001
**CRP**	0.16 (0.15, 0.18)	0.19 (0.17, 0.21)	0.21 (0.19, 0.23)	0.30 (0.26, 0.33)	0.22 (0.20, 0.23)	< 0.001
**Prolactin**	13.21(12.76, 13.67)	12.62 (12.26, 12.97)	12.19 (11.85, 12.53)	12.62 (12.24, 13.00)	12.66 (12.47, 12.85)	0.011
**SC** ^**b**^ **(M/ml)**	55.72 (53.74, 57.69)	52.85 (51.15, 54.55)	54.39 (52.62, 56.16)	50.25 (48.50, 51.99)	53.30 (52.40, 54.20)	< 0.001
**TSM** ^**b**^ **(%)**	65.29 (64.66, 65.92)	65.23 (64.57, 65.88)	64.98 (64.31, 65.66)	64.86 (64.20, 65.51)	65.09 (64.76, 65.41)	0.299
**PRM** ^**b**^ **(%)**	46.00 (45.31, 46.70)	46.21 (45.50, 46.93)	46.44 (45.71, 47.17)	46.04 (45.32, 46.77)	46.18 (45.82, 46.53)	0.832
**NSM** ^**b**^ **(%)**	68.40 (67.74, 69.06)	67.19 (66.53, 67.85)	67.03 (66.35, 67.70)	66.25 (65.58, 66.92)	67.22 (66.88, 67.55)	< 0.001

BMI: Body Mass Index, HDL: High Density Lipoprotein, LDL: Low Density Lipoprotein, Cholesterol Ratio: Cholesterol/HDL, CRP: C-Reactive Protein, SC: Sperm Concentration, TSM: Total Sperm Motility, PRM: Progressive Motility, NSM: Normal Sperm Morphology.

^a^ by one-way analysis of variance (ANOVA).

^b^ the *p* value of SC, TSM, PRM, NSM are 0.005, 0.442, 0.452, <0.001, respectively after adjustment for age, triglyceride, cholesterol, CRP, prolactin and smoking duration (years) by linear regression models.

^c^ the Bonferroni corrected values of *p < 0*.*008* were considered statistically significant.

**Table 2:** Age and anthropometric indexes by semen parameters.

Characteristics	Quartile	Concentration (M/ml) median (*n* = 7630) Mean (95% CI for mean)	% Total Motility median (*n* = 7613) Mean (95% CI for mean)	% Progressive Motility median (*n* = 7601) Mean (95% CI for mean)	% Normal Morphology median (*n* = 7624) Mean (95% CI for mean)
**Age** ^**a**^ **(years)**	Q1: <29	55.34 (53.39, 57.30)	67.56 (66.94, 68.17)	48.23 (47.50, 48.96)	69.45 (68.76, 70.14)
	Q2: 29–30	53.84 (52.01, 55.66)	66.84 (66.16, 67.52)	47.59 (46.82, 48.35)	68.70 (68.00, 69.40)
	Q3: 31–33	51.89 (50.17, 53.61)	65.18 (64.58, 65.78)	46.33 (45.66, 47.01)	66.87 (66.25, 67.49)
	Q4: ≥34	52.60 (50.89, 54.31)	61.719 (61.05, 62.39)	43.33 (42.64, 44.01)	64.64 (64.00, 65.29)
	*p* ^*b*^, *adjusted p*	0.015, 0.004	<0.001, <0.001	<0.001, <0.001	<0.001, <0.001
**BMI** ^**a**^ **(kg/m** ^**2**^)	Q1: <21.60	55.72 (53.74, 57.69)	65.29 (64.66, 65.92)	46.00 (45.31, 46.70)	68.40 (67.74, 69.06)
	Q2: 21.60–23.48	52.85 (51.15, 54.55)	65.23 (64.57, 65.88)	46.21 (45.50, 46.93)	67.19 (66.53, 67.85)
	Q3: 23.49–25.61	54.39 (52.62, 56.16)	64.89 (64.31, 65.66)	46.44 (45.71, 47.17)	67.03 (66.35, 67.70)
	Q4: ≥25.62	50.25 (48.50, 51.99)	64.86 (64.20, 65.51)	46.04 (45.32, 46.77)	66.25 (65.58, 66.92)
	*p* ^*b*^, *adjusted p*	<0.001, 0.005	0.299, 0.403	0.832, 0.488	<0.001, <0.001
**Waist circumference (cm)**	Q1: <75.00	55.05 (53.04, 57.06)	65.66 (64.97, 66.34)	45.80 (45.06, 46.55)	68.29 (67.57, 69.01)
	Q2: 75.00–79.00	54.39 (52.50, 56.28)	64.92 (64.24, 65.60)	46.24 (45.48, 46.99)	67.01 (66.30, 67.73)
	Q3: 80.00–85.00	54.03 (52.30, 55.75)	65.21 (64.59, 65.84)	46.41 (45.74, 47.09)	67.38 (66.75, 68.01)
	Q4: ≥86	50.29 (48.65, 51.94)	64.73 (64.10, 65.36)	46.246 (45.56, 46.94)	66.41 (65.79, 67.04)
	*p* ^*b*^, *adjusted p*	<0.001, 0.685	0.108, 0.939	0.394, 0.129	0.001, 0.219
**Hip circumference (cm)**	Q1: <92.00	56.11 (53.96, 58.27)	65.67 (64.98, 66.36)	45.85 (45.08, 46.61)	68.22 (67.51, 68.92)
	Q2: 92.00–95.99	52.93 (51.27, 54.59)	64.90 (64.25, 65.55)	46.10 (45.39, 46.81)	66.95 (66.29, 67.61)
	Q3: 96–99.99	54.67 (52.86, 56.47)	65.06 (64.41, 65.71)	46.37 (45.66, 47.08)	67.65 (66.99, 68.32)
	Q4: ≥100	50.19 (48.54, 51.84)	64.91 (64.28, 65.53)	46.39 (45.71, 47.08)	66.30 (65.66, 66.94)
	*p* ^*b*^, *adjusted p*	<0.001, 0.457	0.185, 0.543	0.258, 0.218	0.001, 0.712
**Waist-to-hip ratio (%)**	Q1: <80	53.89 (51.91, 55.87)	65.25 (64.50, 66.01)	46.41 (45.57, 47.24)	68.238 (67.435, 69.041)
	Q2: 80–83	54.56 (52.75, 56.37)	65.34 (64.72, 65.95)	46.09 (45.42, 46.76)	67.172 (66.537, 67.808)
	Q3: 84–87	53.27 (51.64, 54.91)	65.20 (64.60, 65.80)	46.29 (45.63, 46.96)	67.318 (66.703, 67.934)
	Q4: ≥88	51.50 (49.68, 53.31)	64.64 (63.97, 65.30)	46.05 (45.32, 46.78)	66.416 (65.757, 67.074)
	*p* ^*b*^, *adjusted p*	0.035, 0.991	0.178, 0.383	0.660, 0.853	0.002, 0.579
**Waist-to-height ratio (%)**	Q1: <44	54.62 (52.74, 56.51)	65.46 (64.82, 66.10)	45.93 (45.23, 46.63)	68.00 (67.33, 68.67)
	Q2: 44–46	53.72 (51.99, 55.46)	65.28 (64.63, 65.93)	46.74 (46.02, 47.46)	67.21 (66.54, 67.89)
	Q3: 47–49	53.71 (51.94, 55.48)	65.25 (64.59, 65.92)	45.92 (45.21, 46.64)	66.97 (66.29, 67.65)
	Q4: ≥50	51.21 (49.39, 53.03)	64.48 (63.79, 65.11)	46.20 (45.48, 46.93)	66.72 (66.06, 67.37)
	*p* ^*b*^, *adjusted p*	0.012, 0.052	0.038, 0.735	0.997, 0.529	0.007, 0.004
**Body fat (%)**	Q1: <19.80	54.36 (52.42, 56.31)	64.62 (63.96, 65.28)	45.59 (44.86, 46.31)	67.63 (66.95, 68.31)
	Q2: 19.80–23.29	53.26 (51.55, 54.97)	64.88 (64.23, 65.53)	45.80 (45.09, 46.50)	67.13 (66.47, 67.79)
	Q3: 23.30–26.79	54.32 (52.50, 56.14)	65.54 (64.89, 66.20)	46.79 (46.07, 47.51)	67.10 (66.43, 67.77)
	Q4: ≥26.80	51.30 (49.55, 53.04)	65.33 (64.69, 65.97)	46.55 (45.84, 47.26)	67.03 (66.37, 67.69)
	*p* ^*b*^, *adjusted p*	0.048, 0.846	0.062, 0.209	0.018, 0.403	0.232, 0.057
**Smoking duration** ^**a**^ **(years)**	Q1: 0	57.47 (54.08, 62.85)	65.08 (63.22, 66.95)	45.79 (45.33, 46.25)	67.70 (65.49, 69.91)
	Q2: <3	53.94 (52.32, 56.32)	64.58 (64.16, 65.01)	44.18 (42.02, 46.34)	67.04 (66.61, 67.47)
	Q3: 3–20	53.46 (51.56, 54.61)	66.12 (65.29, 66.95)	47.07 (46.13, 48.02)	67.44 (66.55, 68.34)
	Q4: ≥20	52.01 (50.04, 53.99)	65.84 (65.15, 66.52)	46.88 (46.11, 47.66)	67.49 (66.79, 68.19)
	*p* ^*b*^, *adjusted p*	0.132, 0.036	0.001, 0.275	0.002, 0.667	0.328, 0.556

the *p* values are calculated by one-way analysis of variance (ANOVA).

the adjusted *p* values are adjustment for age, BMI, triglyceride, cholesterol, CRP, prolactin and smoking duration (years) by linear regression models.

^a^ the adjusted *p* values are adjustment for age, BMI, triglyceride, cholesterol, CRP, prolactin and smoking duration (years) but excluded itself by linear regression models.

^b^ the Bonferroni corrected values of *p < 0*.*008* were considered statistically significant.

## Results

The mean age of the participants in this cross-sectional study was 31.75 years (range, 18 to 75 years), with a mean height of 172.21 cm and a mean body weight of 70.65 kg. The mean BMI, waist circumference, hip circumference, waist-to-hip ratio, waist-to-height ratio and body fat percentage were 23.79 ± 3.30 kg/m^2^, 81.06 ± 8.52 cm, 96.24 ± 6.44 cm, 0.84 ± 0.14, 0.47 ± 0.05 and 23.47% ± 5.49%, the means of sperm concentration, total sperm motility, progressive motility and normal sperm morphology were 53.3 M/ml, 65.09%, 46.18% and 67.22% (Table 1), and the prevalence of abnormal semen quality showed 9.6%, 4.8%, 19.8% and 0.8%, respectively.

Total sperm motility, progressive motility, normal sperm morphology and sperm concentration were all statistically inversely correlated with increasing age (Table 2, simple linear regression; *p* < 0.001, *p* < 0.001, *p* < 0.001, *p* = 0.004). The sperm concentration of each upper quartile anthropometric index was lower than that of the lower quartile index. The mean sperm concentration value of BMI had significantly negative correlations (simple linear regression; *p* = 0.005) with each quartile of obesity and smoking duration had nominally significantly reverse association with the mean sperm concentration (simple linear regression; *p* = 0.036). Normal sperm morphology had a statistically inverse correlation with BMI and waist-to-height ratio (simple linear regression; *p* < 0.001, *p* = 0.004). Although other semen parameters including total sperm motility, progressive motility and normal sperm morphology had a reverse trend with each quartile of smoking duration, this was without statistical significance (Table 2, simple linear regression; *p* = 0.275, *p* = 0.667, *p* = 0.556).

To examine the relationships between age, obesity, smoking and semen quality, the semen quality as assessed by the prevalence of abnormal sperm parameters was examined by quartiles of the anthropometric indexes (BMI, waist circumference, hip circumference, waist-to-hip ratio and waist-to-height ratio). A linear association was found between a higher age and an increased incidence of low sperm concentration (Table 3, < 15 M/ml, *p* = 0.011), low total sperm motility (< 40%, *p* < 0.001), low progressive motility (< 32%, *p* < 0.001) and abnormal sperm morphology (< 30%, *p* = 0.002). A higher BMI was associated with a higher incidence of low sperm concentration and abnormal sperm morphology (*p* = 0.020 and *p* = 0.049, respectively). A higher waist circumference was correlated with a higher incidence of low sperm concentration (*p* = 0.038). No correlation was identified between smoking duration and increased incidence of abnormal semen quality (Table 3).

**Table 3:** The associations of abnormal semen quality with age and anthropometric indexes.

Characteristics	Quartile	Concentration < 15 M/ml, *n* (%)	Total Motility < 40%, *n* (%)	Progressive Motility < 32%, *n* (%)	Normal Morphology < 30%, *n* (%)
**Age (years)**	Q1: <29	142 (8.0)	49 (2.8)	295 (16.7)	9 (0.5)
	Q2: 29–30	145 (9.1)	56 (3.7)	266 (16.7)	8 (0.5)
	Q3: 31–33	207 (10.0)	76 (3.7)	391 (19.0)	11 (0.5)
	Q4: ≥34	242 (11.0)	183(8.4)	558 (25.6)	30 (1.4)
	*p* ^a^	0.011	<0.001	<0.001	0.002
**BMI (kg/m** ^**2**^)	Q1: <21.60	170 (8.9)	85 (4.5)	362 (19.0)	9 (0.5)
	Q2: 21.60–23.48	186 (9.7)	92 (4.8)	376 (19.8)	10 (0.5)
	Q3: 23.49–25.61	164 (8.6)	96 (5.0)	375 (19.7)	17 (0.9)
	Q4: ≥25.62	216 (11.3)	91 (4.8)	397 (20.9)	22 (1.2)
	*p* ^a^	0.020	0.869	0.537	0.049
**Waist circumference (cm)**	Q1: <75.00	130 (8.0)	65 (4.0)	311 (19.2)	9 (0.6)
	Q2: 75.00–79.00	172 (9.6)	97 (5.4)	373 (20.9)	15 (0.8)
	Q3: 80.00–85.00	206 (9.8)	100 (4.8)	390 (18.6)	16 (0.8)
	Q4: ≥86.00	227 (10.9)	100 (4.8)	428 (20.6)	18 (0.9)
	*p* ^a^	0.038	0.282	0.201	0.721
**Hip circumference (cm)**	Q1: <92.00	137 (8.4)	72 (4.4)	315 (19.3)	9 (0.6)
	Q2: 92.00–95.99	181 (9.4)	93 (4.8)	390 (20.2)	10 (0.5)
	Q3: 96–99.99	187 (9.6)	99 (5.1)	379 (19.6)	14 (0.7)
	Q4: ≥100	230 (11.0)	98 (4.7)	418 (20.1)	25 (1.2)
	*p* ^a^	0.057	0.796	0.899	0.052
**Waist-to-hip ratio (%)**	Q1: <80	135 (9.5)	72 (5.1)	278 (19.6)	11 (0.8)
	Q2: 80–83	200 (9.3)	95 (4.4)	435 (20.4)	17 (0.8)
	Q3: 84–87	204 (9.6)	92 (4.3)	417 (19.6)	14 (0.7)
	Q4: ≥88	196 (10.3)	103 (5.4)	372 (19.6)	16 (0.8)
	*p* ^a^	0.774	0.325	0.893	0.920
**Waist-to-height ratio (%)**	Q1: <44	165 (8.7)	87 (4.6)	368 (19.4)	12 (0.6)
	Q2: 44–46	186 (9.9)	84 (4.5)	367 (19.5)	13 (0.7)
	Q3: 47–49	180 (9.5)	91 (4.8)	381 (20.2)	17 (0.9)
	Q4: ≥50	204 (10.5)	100 (5.2)	386 (20.1)	16 (0.8)
	*p* ^a^	0.266	0.735	0.905	0.764
**Body fat (%)**	Q1: <19.80	181 (9.7)	98 (5.3)	386 (20.8)	14 (0.8)
	Q2: 19.80–23.29	181 (9.4)	99 (5.1)	400 (20.8)	11 (0.6)
	Q3: 23.30–26.79	183 (9.6)	83 (4.4)	353 (18.6)	16 (0.8)
	Q4: ≥26.80	191 (9.9)	83 (4.3)	367 (19.2)	16 (0.8)
	*P* ^a^	0.943	0.375	0.213	0.745
**Smoking duration (years)**	Q1: 0	13 (6.9)	6 (3.2)	40 (21.2)	1 (0.5)
	Q2: <3	441 (9.5)	246 (5.3)	946 (20.4)	33 (0.7)
	Q3: 3–20	101 (9.6)	33 (3.1)	185 (17.7)	8 (0.8)
	Q4: ≥20	181 (10.5)	79 (4.6)	337 (19.6)	16 (0.9)
	*P* ^a^	0.078	0.097	0.122	0.197

^a^ by the Chi-square test.

The obese men with the highest BMI quartile had a 1.305-fold higher odds ratio of a low sperm concentration (Table 4, 95% confidence interval (CI): 1.056–1.613) as compared with the lowest BMI quartile. In addition, waist circumference had a 1.397-fold higher odds ratio (95% CI: 1.114–1.751), hip circumference a 1.350-fold higher odds ratio (95% CI: 1.081–1.686), and waist-to-height ratio a 1.241-fold higher odds ratio (95% CI: 1.000–1.539) of oligospermia. Men with the highest BMI quartile had a 2.463-fold higher odds ratio (95% CI: 1.131–5.362) and hip circumference had a 2.185-fold higher odds ratio (95% CI: 1.017–4.695) of abnormal sperm morphology as compared with each of the lowest anthropometric index quartiles.

**Table 4:** The odds ratio (OR) and 95% confidence interval (CI) of abnormal semen quality with age and anthropometric indexes.

Characteristics	Quartile	Concentration < **15 M**/ml, OR (95% CI)	Total Motility < 40%, OR (95% CI)	Progressive Motility < 32%, OR (95% CI)	Normal Morphology < 30%, OR (95% CI)
**Age (years)**	Q1: <29	Reference	Reference	Reference	Reference
	Q2: 29–30	1.15 (0.90, 1.46)	1.28 (0.87, 1.89)	1.00 (0.84, 1.20)	0.99 (0.38, 2.57)
	Q3: 31–33	**1.28 (1.02, 1.60)**	1.35 (0.94, 1.94)	1.17 (0.99, 1.38)	1.05 (0.44, 2.54)
	Q4: ≥34	**1.42 (1.15, 1.77)**	**3.22 (2.33, 4.44)**	**1.72 (1.47, 2.01)**	**2.72 (1.29, 5.75)**
**BMI (kg/m** ^**2**^)	Q1: <21.60	Reference	Reference	Reference	Reference
	Q2: 21.60–23.48	1.10 (0.89, 1.37)	1.09 (0.80, 1.47)	1.05 (0.89, 1.23)	1.11 (0.45, 2.74)
	Q3: 23.49–25.61	0.96 (0.77, 1.20)	1.14 (0.84, 1.53)	1.05 (0.89, 1.23)	1.90 (0.84, 4.26)
	Q4: ≥25.62	**1.31 (1.06, 1.61)**	1.08 (0.79, 1.46)	1.13 (0.96, 1.32)	**2.46 (1.13, 5.36)**
**Waist circumference (cm)**	Q1: <75.00	Reference	Reference	Reference	Reference
	Q2: 75.00–79.00	1.22 (0.96, 1.55)	1.38 (0.99, 1.908)	1.11 (0.94, 1.32)	1.52 (0.66, 3.48)
	Q3: 80.00–85.00	1.241 (0.99,1.56)	1.19 (0.87, 1.64)	0.96 (0.81, 1.13)	1.37 (0.60, 3.11)
	Q4: ≥86.00	**1.40 (1.11, 1.75)**	1.21 (0.88, 1.66)	1.09 (0.93, 1.28)	1.56 (0.70, 3.47)
**Hip circumference (cm)**	Q1: <92.00	Reference	Reference	Reference	Reference
	Q2: 92.00–95.99	1.13 (0.90, 1.43)	1.10 (0.80, 1.50)	1.06 (0.90, 1.25)	0.94 (0.38, 2.32)
	Q3: 96.00–99.99	1.17 (0.93, 1.47)	1.17 (0.86, 1.60)	1.02 (0.86, 1.20)	1.32 (0.57, 3.05)
	Q4: ≥100	**1.35 (1.08, 1.69)**	1.07 (0.78, 1.46)	1.05 (0.89, 1.23)	**2.19 (1.02, 4.70)**
**Waist-to-hip ratio (%)**	Q1: <80	Reference	Reference	Reference	Reference
	Q2: 80–83	0.98 (0.78, 1.24)	0.87 (0.64, 1.19)	1.00 (0.84, 1.19)	1.03 (0.48, 2.20)
	Q3: 84–87	1.01 (0.80, 1.27)	0.85 (0.62, 1.16)	1.05 (0.90, 1.23)	0.85 (0.38, 1.87)
	Q4: ≥88	1.09 (0.87, 1.37)	1.07 (0.79, 1.46)	1.00 (0.86, 1.17)	1.08 (0.50, 2.34)
**Waist-to-height ratio (%)**	Q1: <44	Reference	Reference	Reference	Reference
	Q2: 44–46	1.15 (0.92, 1.43)	0.97 (0.72, 1.32)	1.01 (0.86, 1.18)	1.09 (0.50, 2.40)
	Q3: 47–49	1.11 (0.90, 1.39)	1.06 (0.78, 1.43)	1.05 (0.90, 1.23)	1.43 (0.68, 3.01)
	Q4: ≥50	**1.24 (1.00, 1.54)**	1.14 (0.85, 1.53)	1.04 (0.90, 1.22)	1.31 (0.62, 2.78)
**Body fat (%)**	Q1: <19.80	Reference	Reference	Reference	Reference
	Q2: 19.80–23.29	0.96 (0.77, 1.19)	0.97 (0.73, 1.29)	0.99 (0.85, 1.17)	0.76 (0.34, 1.67)
	Q3: 23.30–26.79	0.98 (0.79, 1.22)	0.82 (0.61, 1.11)	0.87 (0.74, 1.02)	1.12 (0.54, 2.29)
	Q4: ≥26.80	1.02 (0.83, 1.27)	0.81 (0.60, 1.10)	0.90 (0.77, 1.06)	1.11 (0.54, 2.28)
**Smoking duration (years)**	Q1: 0	Reference	Reference	Reference	Reference
	Q2: <3	1.42 (0.80, 2.51)	1.71 (0.75, 3.89)	0.96 (0.67, 1.36)	1.35 (0.18, 9.88)
	Q3: 3–20	1.44 (0.79, 2.62)	0.99 (0.41, 2.40)	0.80 (0.54, 1.17)	1.44 (0.18, 11.60)
	Q4: ≥20	1.58 (0.88, 2.83)	1.46 (0.63, 3.40)	0.91 (0.63, 1.31)	1.75 (0.23, 13.31)

## Discussion

Our study was the first to report the largest cross-sectional study in a single institute that enrolled subjects from a general population and used standardized protocols of a regular medical screening program including measurements of body size and biochemical data to assess anthropometric characteristics and semen quality. The findings demonstrated that age and obesity were correlated with semen quality.

Age was statistically inversely correlated with all of the measured sperm parameters (total sperm motility, progressive motility, normal sperm morphology and sperm concentration; *p <* 0.001, *p <* 0.001, *p <* 0.001 *and p =* 0.004), and abnormal incidences (*p =* 0.011, *p <* 0.001, *p <* 0.001 *and p =* 0.002), indicating that an increasing age was associated with poorer semen quality and higher incidences of abnormal sperm parameters. Two recent review articles concluded that semen quality, including volume, sperm motility and morphology, deteriorated with increased age; however, the results concerning sperm concentration were inconsistent [17, 18]. However, most previous studies have included a smaller number of cases and a narrower age range as compared with the present study.

Two recent meta-analyses [6, 8] explored the relationship between obesity and semen production; however, the conclusions of these studies were inconsistent. One of the studies reviewed 31 studies with 5 included in the pooled meta-analysis, a total of 6,793 men, and found no significant association between BMI and sperm parameters [6]. Another study of Sermondade reported that the overweight men had significantly higher levels of oligozoospermia or azoospermia as compared with the men with a normal BMI [8]. Neither of these meta-analyses showed a linear relationship between BMI and sperm concentration or total sperm count, but had contrasting conclusions with regards to obesity and the incidence of an abnormal sperm concentration. A possible explanation is that most of the enrolled subjects had subfertility or infertility and BMI was self-reported. In addition, both of these meta-analyses were limited in only assessing general obesity with BMI, and so whether or not central obesity was correlated with semen quality could not be clarified. Moreover, some studies of other anthropometric measures have reported that abdominal fat may be a risk factor for several diseases independent of BMI [19].

Several studies have discussed the association between central obesity or other measures of fat distribution and sperm parameters [4, 5, 20]. Higher wait circumference and hip circumference have been shown to be associated with a lower total sperm count, total motile sperm count and progressive motile sperm count [5]. There is evidence that sperm concentration and total motile sperm count are detrimentally affected by a high BMI and waist circumference [4]. Study using data from the Longitudinal Investigation of Fertility and Environment has demonstrated that ejaculate volume and total sperm count have inverse linear associations with waist circumference [20]. The results of our current study showed that obesity was inversely correlated with sperm concentration and normal sperm morphology both in terms of general obesity assessed with BMI (both *p* < 0.001) and central obesity assessed with waist circumference (*p* < 0.001; *p* = 0.001) and hip circumference (*p* < 0.001; *p* = 0.001) before the co-factors being adjusted. However only BMI illustrated significantly statistical relationship (*p* = 0.005; *p* < 0.001) after adjusting related factors. Meanwhile the general obesity as assessed by BMI displayed significantly positive correlations with incidence of oligospermia and abnormal sperm morphology (*p* = 0.020; *p* = 0.049), but also the other anthropometric indexes of waist circumference (*p* = 0.038) and hip circumference (*p* = 0.057; *p* = 0.052) declared the similar trend.

Several studies have proposed waist-to-height ratio as another proxy for central obesity, correcting the waist circumference for the height of the individuals. A systematic review suggested that waist-to-height ratio is a more useful global clinical screening tool than waist circumference [21], and a meta-analysis supported the use of waist-to-height ratio in identifying adults at increased cardiometabolic risk and concluded that waist-to-height ratio appeared to be more useful in Asian than in non-Asian populations [22]. In addition, population-based cross-sectional studies reported that waist-to-height ratio is the best screening tool for detecting cardiometabolic risk factors in Chinese populations [23, 24]. However, in the current study, the waist-to-height ratio of anthropometric index showed a negative trend with sperm concentration with only borderline significance (*p* = 0.052) and a statistically inverse association with normal sperm morphology (*p* = 0.004). Therefore, it was not as powerful as BMI, waist circumference and hip circumference in the prediction of sperm parameters.

The etiology of the relationship between adiposity and sperm production is complex and unclear. Overweight and obesity, and particularly central obesity, have been shown to affect the GnRH-FSH/LH pulse, which may impair Leydig and Sertoli cell functions and thus interfere with the release of sex hormones and production of mature of sperm [25, 26]. The inverse association between a high BMI and sperm parameters found in this study support the findings of the aforementioned studies. However, as the majority of previous studies have focused only on BMI as the predominant measure of adiposity and not on other anthropometric indexes, our current study is the second to show associations between hip circumference, waist-to-height ratio and sperm parameters [5], and is the first to focus on the relationship between waist-to-height ratio and semen quality in a large number of cases within the general population at a single institute.

In this study, we found inverse associations between obesity and sperm parameters including sperm concentration and normal sperm morphology. This is in accordance with the results of previous studies [3, 27, 28], all of which concluded that a high BMI (> 25 kg/m^2^) was significantly associated with reduced sperm concentration and a lower percentage of normal spermatozoa without significance. The meta-analysis by Sermondade, Faure [8] demonstrated that the underweight (BMI < 18.5 kg/m^2^) and obese (BMI > 30 kg/m^2^) groups had higher risks of oligozoospermia or azoospermia. However, the results of the current study did not show a similar relationship, but rather a positive linear association. It is possible that the number of underweight subjects (2.21% of 13077 subjects) was too low in Sermondade *et al*.’s study to illustrate the real correlation between obesity and a lower total sperm count. Our report showed that only sperm concentration was nominally inversely correlated with increased smoking duration, which was similar with Collodel’s study [29]. Although sperm concentration, motility and/or morphology are often reduced as compared with the results observed in nonsmokers, they often remained within the normal range. However the 2012 ASRM (American Society for Reproduction Medicine) Practice Committee concluded that semen parameters and results of sperm function tests are 22% poorer in smokers than in nonsmokers with a dose-dependent effect, but smoking has not yet been conclusively shown to reduce male fertility [30]. The result of our current study was promising but requiring further confirmation/replication in an independent study, and the questionnaire of the smoking intensity would be considered to explore the real association between smoking and semen quality in future.

There were some limitations to this study. We lacked data on semen volume and total sperm count due to the protocol of the physical health examination. It was regretful that we did not have detailed records of endocrine levels to assist in achieving a greater understanding of the mechanisms behind the association between semen quality and obesity. Some parameters of our study were relatively gross measures of fertility, and we did not attempt to examine functional parameters such as the DNA fragmentation index and seminal oxidative stress. Studies using the DNA fragmentation index as a measure of genetic quality have reported statistically significantly positive correlations between BMI and DFI [31, 32], indicating that obesity may still reduce the semen quality even if the sperm count and other sperm parameters remain unchanged. A higher BMI has been shown to have a statistically significant association with increased seminal reactive oxidative stress, and that elevated semen inflammation detected increased seminal plasma polymorphonuclear leukocytes (PMNs) elastase and neopterin concentrations in 81 men [33]. A positive correlation between BMI and sperm DNA fragmentation has been found in two mouse models of obesity [34], suggesting that sperm chromatin integrity is positively associated with BMI. Another longitudinal cross sectional study of Håkosen [35] ever concluded that obesity was associated with poor semen quality and altered reproductive hormonal profile. Meanwhile the group with the largest weight loss had a statistically significant increase in total sperm count and normal sperm morphology. However they observed no significant difference in DFI from baseline to follow-up.

Further studies including those with the abovementioned sperm molecular markers should be designed to explore the mechanism between obesity and semen quality. Meanwhile, the enrolled subjects were centralized in the relatively young populations, although the age distribution of the study varied from 18 to 75 years. We could hypothesize the associated trend between age and semen quality in a larger size population with the limitation of the majority subjects being in the relatively young age distribution. In addition, the participants had to pay a fee (∼US$100) to participate in the medical screening program, which, although acceptable for the middle classes of the general population in Taiwan, may exclude poorer populations. As the participants had only been asked to have more than 3 days of abstinence, but longer abstinence duration has not been recorded in the data of MJ Health Management Institution, and therefore the effect of abstinence time could not be performed. Furthermore, as this was a large sample size study, it was difficult to perform on-site collection of semen samples. Therefore, semen samples were collected using home-collection kits. As the semen samples were requested to be sent to the laboratory within one hour, the quality may not be equal to that resulting from on-site collection. Ultimately the effect of environmental chemicals like PFCs and lipophilics food should be took into consideration for comprehensive association between obesity and semen quality owing to the expectedly increased intake of above chemicals combined with food in the obese subjects, however the above data and records were unavailable on account of the original study design.

## Conclusions

The results of this study showed that increased age was statistically inversely associated with semen quality, and that increased adiposity was significantly negatively correlated with sperm concentration and normal sperm morphology in a large population of healthy Taiwanese men.

## References

[1] Pasquali R, Patton L, Gambineri A. Obesity and infertility. Current opinion in endocrinology, diabetes, and obesity. 2007;14: 482–487. 17982356 10.1097/MED.0b013e3282f1d6cb

[2] Ramlau-Hansen CH, Thulstrup AM, Nohr EA, Bonde JP, Sorensen TI, Olsen J. Subfecundity in overweight and obese couples. Hum Reprod. 2007;22: 1634–1637. 17344224 10.1093/humrep/dem035

[3] Jensen TK, Andersson AM, Jorgensen N, Andersen AG, Carlsen E, Petersen JH, et al Body mass index in relation to semen quality and reproductive hormones among 1,558 Danish men. Fertility and sterility. 2004;82: 863–870. 15482761 10.1016/j.fertnstert.2004.03.056

[4] Hammiche F, Laven JS, Twigt JM, Boellaard WP, Steegers EA, Steegers-Theunissen RP. Body mass index and central adiposity are associated with sperm quality in men of subfertile couples. Hum Reprod. 2012;27: 2365–2372. 10.1093/humrep/des177 22693175

[5] Fejes I, Koloszar S, Szollodsi J, Zavaczki Z, Pal A. Is semen quality affected by male body fat distribution? Andrologia. 2005;37: 155–159. 16266392 10.1111/j.1439-0272.2005.00671.x

[6] MacDonald AA, Herbison GP, Showell M, Farquhar CM. The impact of body mass index on semen parameters and reproductive hormones in human males: a systematic review with meta-analysis. Human reproduction update. 2010;16: 293–311. 10.1093/humupd/dmp047 19889752

[7] Macdonald AA, Stewart AW, Farquhar CM. Body mass index in relation to semen quality and reproductive hormones in New Zealand men: a cross-sectional study in fertility clinics. Hum Reprod. 2013;28: 3178–3187. 10.1093/humrep/det379 24129611

[8] Sermondade N, Faure C, Fezeu L, Shayeb AG, Bonde JP, Jensen TK, et al BMI in relation to sperm count: an updated systematic review and collaborative meta-analysis. Human reproduction update. 2013;19: 221–231. 10.1093/humupd/dms050 23242914 PMC3621293

[9] Palmer NO, Bakos HW, Fullston T, Lane M. Impact of obesity on male fertility, sperm function and molecular composition. Spermatogenesis. 2012;2: 253–263. 23248766 10.4161/spmg.21362 PMC3521747

[10] Colaci DS, Afeiche M, Gaskins AJ, Wright DL, Toth TL, Tanrikut C, et al Men's body mass index in relation to embryo quality and clinical outcomes in couples undergoing in vitro fertilization. Fertility and sterility. 2012;98: 1193–1199 e1191. 10.1016/j.fertnstert.2012.07.1102 22884013 PMC3478419

[11] Stéphanie Belloc, Martine Cohen-Bacrie, Edouard Amar, Vincent Izard, Moncef Benkhalifa, Alain Dalléac, et al. High body mass index has a deleterious effect on semen parameters except morphology: results from a large cohort study Fertility and sterility. 2014; 10.1016/j.fertnstert.2014.07.1212 25225071

[12] WHO. World Health Organization. WHO Laboratory Manual for the Examination and Processing of Human Semen, 5th edn. Geneva, Switzerland: WHO Press, 2010. 2010;

[13] Menkveld R, Stander FS, Kotze TJ, Kruger TF, van Zyl JA. The evaluation of morphological characteristics of human spermatozoa according to stricter criteria. Hum Reprod. 1990;5: 586–592. 2394790 10.1093/oxfordjournals.humrep.a137150

[14] consultation We. Appropriate body-mass index for Asian populations and its implications for policy and intervention strategies. Lancet. 2004;363: 157–163. 14726171 10.1016/S0140-6736(03)15268-3

[15] Deurenberg P, Deurenberg-Yap M, Guricci S. Asians are different from Caucasians and from each other in their body mass index/body fat per cent relationship. Obes Rev. 2002;3: 141–146. 12164465 10.1046/j.1467-789x.2002.00065.x

[16] Pan W-H, Yeh W-T. How to define obesity? Evidence-based multiple action points for public awareness, screening, and treatment: an extension of Asian-Pacific recommendations. Asia Pac J Clin Nutr. 2008;17: 370–374. 18818155

[17] Kuhnert B, Nieschlag E. Reproductive functions of the ageing male. Human reproduction update. 2004;10: 327–339. 15192059 10.1093/humupd/dmh030

[18] Sartorius GA, Nieschlag E. Paternal age and reproduction. Human reproduction update. 2010;16: 65–79. 10.1093/humupd/dmp027 19696093

[19] Janssen I, Katzmarzyk PT, Ross R. Waist circumference and not body mass index explains obesity-related health risk. The American journal of clinical nutrition. 2004;79: 379–384. 14985210 10.1093/ajcn/79.3.379

[20] Eisenberg ML, Kim S, Chen Z, Sundaram R, Schisterman EF, Buck Louis GM. The relationship between BMI and waist circumference on semen quality: data from the LIFE study. Human Reproduction. 2014;29: 193–200. 10.1093/humrep/det428 24306102 PMC3896223

[21] Browning LM, Hsieh SD, Ashwell M. A systematic review of waist-to-height ratio as a screening tool for the prediction of cardiovascular disease and diabetes: 0·5 could be a suitable global boundary value. Nutr Res Rev. 2010;23: 247–269. 10.1017/S0954422410000144 20819243

[22] Savva SC, Lamnisos D, Kafatos AG. Predicting cardiometabolic risk: waist-to-height ratio or BMI. A meta-analysis. Diabetes, metabolic syndrome and obesity: targets and therapy. 2013;6: 403–419. 10.2147/DMSO.S34220 24179379 PMC3810792

[23] Shao J, Yu L, Shen X, Li D, Wang K. Waist-to-height ratio, an optimal predictor for obesity and metabolic syndrome in Chinese adults. The journal of nutrition, health & aging. 2010;14: 782–785. 10.1007/s12603-010-0106-x PMC12880338 21085910

[24] Zhang Z-q, Deng J, He L-p, Ling W-h, Su Y-x, Chen Y-m. Comparison of various anthropometric and body fat indices in identifying cardiometabolic disturbances in Chinese men and women. PLoS One. 2013;8: e70893 10.1371/journal.pone.0070893 23951031 PMC3741370

[25] Hammoud AO, Gibson M, Peterson CM, Meikle AW, Carrell DT. Impact of male obesity on infertility: a critical review of the current literature. Fertility and sterility. 2008;90: 897–904. 10.1016/j.fertnstert.2008.08.026 18929048

[26] Hammoud AO, Wilde N, Gibson M, Parks A, Carrell DT, Meikle AW. Male obesity and alteration in sperm parameters. Fertility and sterility. 2008;90: 2222–2225. 10.1016/j.fertnstert.2007.10.011 18178190

[27] Paasch U, Grunewald S, Kratzsch J, Glander HJ. Obesity and age affect male fertility potential. Fertility and sterility. 2010;94: 2898–2901. 10.1016/j.fertnstert.2010.06.047 20667533

[28] Qin DD, Yuan W, Zhou WJ, Cui YQ, Wu JQ, Ga ES. Do reproductive hormones explain the association between body mass index and semen quality? Asian J Androl. 2007;9: 827–834. 17968470 10.1111/j.1745-7262.2007.00268.x

[29] Collodel G, Capitani S, Pammolli A, Giannerini V, Geminiani M, Moretti E. Semen quality of male idiopathic infertile smokers and nonsmokers: an ultrastructural study. Journal of andrology. 2010;31: 108–113. 10.2164/jandrol.109.007773 19745220

[30] Practice Committee of the American Society for Reproductive M, Pfeifer S, Fritz M, Goldberg J, McClure RD, Thomas M, et al Smoking and infertility: a committee opinion. Fertility and sterility. 2012;98: 1400–1406. 10.1016/j.fertnstert.2012.07.1146 22959451

[31] Chavarro JE, Toth TL, Wright DL, Meeker JD, Hauser R. Body mass index in relation to semen quality, sperm DNA integrity, and serum reproductive hormone levels among men attending an infertility clinic. Fertility and sterility. 2010;93: 2222–2231. 10.1016/j.fertnstert.2009.01.100 19261274 PMC2864498

[32] Kort HI, Massey JB, Elsner CW, Mitchell-Leef D, Shapiro DB, Witt MA, et al Impact of body mass index values on sperm quantity and quality. Journal of andrology. 2006;27: 450–452. 16339454 10.2164/jandrol.05124

[33] Tunc O, Bakos HW, Tremellen K. Impact of body mass index on seminal oxidative stress. Andrologia. 2011;43: 121–128. 10.1111/j.1439-0272.2009.01032.x 21382066

[34] Duale N, Steffensen IL, Andersen J, Brevik A, Brunborg G, Lindeman B. Impaired sperm chromatin integrity in obese mice. Andrology. 2014;2: 234–243. 10.1111/j.2047-2927.2013.00178.x 24459046

[35] Hakonsen LB, Thulstrup AM, Aggerholm AS, Olsen J, Bonde JP, Andersen CY, et al Does weight loss improve semen quality and reproductive hormones? Results from a cohort of severely obese men. Reproductive health. 2011;8: 24 10.1186/1742-4755-8-24 21849026 PMC3177768

